# TRAINING FOR CAREGIVER OF OLDER ADULTS IN ECUADOR: REPORT ON PRESENTIAL, DISTANCE, AND HYBRID MODALITIES

**DOI:** 10.1093/geroni/igad104.2397

**Published:** 2023-12-21

**Authors:** Jonathan Guillemot, Samantha Saenz, Maria Augusta Vélez Landazuri

**Affiliations:** Universidad San Francisco de Quito, Quito, Pichincha, Ecuador; Universidad San Francisco de Quito, Quito, Pichincha, Ecuador; Universidad San Francisco de Quito, Quito, Pichincha, Ecuador

## Abstract

Caregiving for older adults in Ecuador is provided by family members, professional caregivers as well as untrained domestic workers. All three groups lack access to theoretical and technical training. The accelerating care drain towards Europe and North America, the fast-paced aging in the region as well as increasing trends of North American older adults migration to Ecuador, increase the need for large scale, quality and affordable caregiver training. We report the methods and outcomes of three modalities of training for caregivers of older adults combining over 100 individuals trained over the course of four years: a traditional presential training, which was offered prior to the COVID-19 pandemic; an online modality via Zoom during the pandemic; and the early outcomes of a novel hybrid modality, combining presential and asynchronous video-based learning. We found that social interaction, including the sharing of personal experiences, was an essential part of the training experience, which translated into long-term social bonds and early stages of solidarity networks between participants. Online synchronous training allowed a broader geographical coverage but failed to contribute to socialization as well as generated challenges related to the learning experience and efficacy. The novel modality offered the broad geographical coverage, lowered the cost and allowed socialization. It however created technical and quality control challenges. Training of caregivers is a key component to promote aging in place and age-friendliness. Our experience and conclusion aim to contribute to the future of training in the Global South as well as in the Global North.

